# *Lutzomyia longipalpis* Presence and Abundance Distribution at Different Micro-spatial Scales in an Urban Scenario

**DOI:** 10.1371/journal.pntd.0003951

**Published:** 2015-08-14

**Authors:** María Soledad Santini, María Eugenia Utgés, Pablo Berrozpe, Mariana Manteca Acosta, Natalia Casas, Paola Heuer, O. Daniel Salomón

**Affiliations:** 1 Centro Nacional de Investigación en Endemo-epidemias (CeNDIE), ANLIS, Ministerio de Salud de la Nación, Buenos Aires, Argentina; 2 Comité Nacional de Investigaciones Científicas y Técnicas (CONICET), Ministerio de Ciencia, Tecnología e Innovación Productiva, Buenos Aires, Argentina; 3 Leishmaniasis Investigation Network of Argentina (ReDILA); 4 Instituto Nacional de Medicina Tropical (INMeT), Ministerio de Salud de la Nación, Puerto Iguazú, Misiones, Argentina; 5 Programa Nacional de Control de Enfermedades Zoonóticas (ProNCEZ), Ministerio de Salud de la Nación, Buenos Aires, Argentina; 6 Laboratorio de Control de Vectores Entomológicos de Importancia Sanitaria (LaCVEIS) Fundación H. A. Barceló, sede Santo Tomé, Corrientes, Argentina; National Institute of Allergy and Infectious Diseases, UNITED STATES

## Abstract

The principal objective of this study was to assess a modeling approach to *Lu*. *longipalpis* distribution in an urban scenario, discriminating micro-scale landscape variables at microhabitat and macrohabitat scales and the presence from the abundance of the vector. For this objective, we studied vectors and domestic reservoirs and evaluated different environmental variables simultaneously, so we constructed a set of 13 models to account for micro-habitats, macro-habitats and mixed-habitats. We captured a total of 853 sandflies, of which 98.35% were *Lu*. *longipalpis*. We sampled a total of 197 dogs; 177 of which were associated with households where insects were sampled. Positive rK39 dogs represented 16.75% of the total, of which 47% were asymptomatic. Distance to the border of the city and high to medium density vegetation cover ended to be the explanatory variables, all positive, for the presence of sandflies in the city. All variables in the abundance model ended to be explanatory, trees around the trap, distance to the stream and its quadratic, being the last one the only one with negative coefficient indicating that the maximum abundance was associated with medium values of distance to the stream. The spatial distribution of dogs infected with *L*. *infantum* showed a heterogeneous pattern throughout the city; however, we could not confirm an association of the distribution with the variables assessed. In relation to *Lu*. *longipalpis* distribution, the strategy to discriminate the micro-spatial scales at which the environmental variables were recorded allowed us to associate presence with macrohabitat variables and abundance with microhabitat and macrohabitat variables. Based on the variables associated with *Lu*. *longipalpis*, the model will be validated in other cities and environmental surveillance, and control interventions will be proposed and evaluated in the microscale level and integrated with socio-cultural approaches and programmatic and village (mesoscale) strategies.

## Author Summary

Visceral leishmaniasis in America is caused by an unicellular organism, *Leishmania infantum* (syn. *chagasi)* that is transmitted by insects belonging to Diptera:Phlebotominae, *Lutzomyia longipalpis* being the principal vector in urban areas. Therefore, the prevention and control of this vector is a sound objective, so as to reduce the probability of contact human-vector and reducing the probability of infection. Therefore, knowing the variables that have an impact and the spatial scale at which these act will allow us to approach an understanding of the dynamic population of the vector and allow us to develop more appropriate strategies of control. Thus, the aim of this study was to assess a modeling approach to *Lu*. *longipalpis* distribution in an urban scenario, discriminating micro-scale landscape variables at microhabitat and macrohabitat scales. For this, we worked in Santo Tomé, Corrientes, Argentina. We observed that the presence of *Lu*. *longipalpis* is defined only by the macrohabitat variables tested, but the abundance is defined by variables of both scales, microhabitat and macrohabitat.

## Introduction

Visceral leishmaniasis (VL) in America is caused by *Leishmania infantum* (syn. *chagasi*). The sandfly *Lutzomyia longipalpis* was incriminated as the most important vector [1] and the domestic dog was involved as the main reservoir, both in urban areas [2–5].

Although *Lu*. *longipalpis* was recorded in Argentina at forest-rural sites in 1951 and 2000 with very few individuals per capture, since 2006 this species has been found in VL urban foci in captures with more than 100 insects per trap in the first focus at the city of Posadas, Province of Misiones, and also present in other cities of northeastern Argentina (provinces of Formosa and Chaco), [5–9]. Salomón et al. [10,11] studied the presence and distribution of *Lu*. *longipalpis* in the province of Corrientes (contiguous to Misiones where Posadas is close to the border between both provinces) to assess the possibility of autochthonous transmission of *L*. *infantum*. This province has an active transmission scenario with canine leishmaniasis cases and vector presence since 2008 [10], even in Santo Tomé, resulting in 16 human cases that have been diagnosed since 2010 till the 20^th^ epidemiological week of 2015 (9 of which were recorded at Santo Tomé, with 3 deaths). Despite canine leishmaniasis was diagnosed in numerous dogs, no systematic rate of infected dogs was performed until this study.

Dynamic epidemiological patterns of transmission are the result of the simultaneous and multi-scale interaction of biotic factors that coexist in heterogeneous epidemiological landscapes [12,13]. In this sense, Real and Biek [14] hypothesize that the spatial context and the geographic landscape contribute to the initial establishment of the disease. It should be noted that the scales from microfocal to regional, although they are inclusive to each other in increasing order, require questions, resolution, data quality, and different analytical tools to support the conclusions appropriate to each scale [13,15]. At a coarse resolution the micro-scale heterogeneity may not be detected, as well as general macro-scale patterns may be overlooked at a fine spatial resolution [16].

Previous studies on leishmaniasis associated *Lu*. *longipalpis* abundance in urban scenarios with the presence of chickens, dogs and/or fruit trees, or Normalized Difference Vegetation Index (NDVI) ranges, which can offer suitable conditions for reproductive success of the vector [17–22]. A study carried out in the city of Posadas, identified microhabitat variables such as *surface of bare soil* or *covered with grass*, *distance from house to watercourse*, *number of plant-pots*, and *number of tree species* as possible contributors to the abundance of vectors in an urban environment [23]. Despite these results, factors associated with the increase in presence and abundance of *Lu*. *longipalpis* in urban environments are only partially understood [24], and the modeling at micro-scale usually explain up to 30% of the variability [25]. The micro-scale is defined by the characteristics of the house and surrounding area, and is the operational scale for focal interventions [15,26]. But when modeling *Lu*. *longipalpis* abundance in Posadas city at this scale, the vector showed different associations between variables recorded at micro-habitat (trap site) and macro-habitat variables (theoretically the smallest homogeneous patch of the variable, instrumentally a buffer area that includes relatively homogeneous surroundings). Further, in this urban setting more than 30%-40% of the sites sampled had *Lu*. *longipalpis* presence while less than 5% had high abundance of the vector, suggesting that the presence and the abundance are modulated by different variables [25]. Therefore, the principal objective of this study was to assess a modeling approach to *Lu*. *longipalpis* distribution in an urban scenario different from Posadas, discriminating micro-scale landscape variables at microhabitat and macrohabitat scales, and the presence from the abundance of the vector, in order to try to improve the explanatory power of the model, and so to contribute to the design of integrated intervention strategies based on the associated variables. The visceral canine leishmaniasis distribution was also analyzed as it was proposed as indicator of transmission or human risk [27–29].

## Materials and Methods

### Area of study

This study was carried out in Santo Tomé City, Corrientes, Argentina (28°33'5.79"S, 56° 2'44.11"W). This city belongs to the ‘Espinal’ ecoregion, Neotropical ecozone [30], and it is situated on the coast of the Uruguay River which determines the border between Argentina and Brazil. Santo Tomé has a stable population of 23,299 inhabitants [31] distributed in approximately 8 km^2^.

### Sampling

The study was conducted from 25 to 27^th^ February 2013. We studied vectors and domestic reservoirs simultaneously. In order to sample the entire urban area, the city was divided into a grid of 600 m^2^ squares (patch), except for the neighborhood ‘Estación’ on the West, where high vector abundance had been reported by a previous study [11], and was divided into 200 m^2^ squares. One domestic unit was selected within each patch using the ‘worst scenario’ criterion [32]. The ‘worst scenario’ is a functional definition to denote a site within the study patch with the greatest probability of sandfly presence due to habitat conditions. ‘Worst scenarios’ are distinguished by the presence of dense vegetation which provides shadow, humidity and detritus; soil rich in organic material and access to blood ingestion without the interference of external light. In the 600 m^2^ patches, minimum and maximum distances between traps settled in different patches were 145 and 472 m respectively; whereas in the 200 m^2^ patches, minimum and maximum distances between traps were 110 and 270 m respectively. The geographic coordinates of all the sites sampled were registered with a Global Positioning System (Garmin eTrex10).

### Entomological sampling

Sandflies were captured with automatic CDC-like light traps, used for the sampling of Phlebotominae in peridomestic environments. Traps were active from approximately 5:30 p.m. to 7:30 a.m., for 3 consecutive rainless nights. Traps were placed 1.5 m above the ground.

All Phlebotominae sandflies were dried and preserved prior to processing. The specimens were cleared with lacto-phenol and identified according to [33] under a microscope (Zeiss, 400x). *Evandromyia cortelezzii* and *Ev*. *sallesi* females cannot be distinguished by their morphology, so specimens collected were included within the *Ev*. *cortelezzii-sallesi* complex.

According to previous studies in urban areas where traps with more than 30 *Lu*. *longipalpis* individuals summed up to the 10–15 percentile, we operatively classified the domestic units into low (<30) and high abundance (>30) [20].

### Climatic data

Maximum (max) and minimum (min) temperatures (T) and relative humidity (RH) were registered during sampling in the trap active period with digital thermo-hygrometers (TFA, Germany) in 17 randomly selected domestic units. During the capture period mean climatic variables were: Tmin mean: 15.42, SE: 1.75; Tmax mean: 31.43, SE: 1.02; RH min mean: 39.46, SE: 5.59; RH max mean: 92.28, SE: 6.55.

### Canine sampling

Dogs from the houses with sandfly traps were blood-sampled by veterinarians, *Dogs house*. We also sampled all dogs in neighboring houses within a 25 m radius, *Dogs neighbours*. The presence of antibodies against *L*. *infantum* by means of the immunochromatographic rK39 technique was done *in situ* (Kalazar Detect Canine Rapid Test; InBios). For each dog, 11 variables were gathered: breed (yes/no), gender, age (years), size (small, medium, large), sterilization (yes/no), night resting place (interior/exterior), unleashed (allowed to wander around, yes/no), moving history (yes/no), repellent use (yes/no), repellent periodicity (months), symptoms (yes/no).

### Ethics statement

The study was conducted according to the ethical regulations for research established by the World Organization for Animal Health (OIE) [34] and with the approval of the ethics committee ‘Comité de Ética de Investigación Clínica’ (CEIC, Office for Human Research Protection, IRB Registration 00001678 –USA; Res. N° 1108–26). All the neighbours that collaborated in the study were informed about the practices and signed an informed consent form.

### Environmental variables

Satellite information to generate the environmental stratification of the city was obtained from a Spot 5 HRG1 J image (spatial resolution, 10 m; March 2013, facilitated by a CONAE-Argentina and CNES-France agreement). The synthetic image was digitally processed in order to convert digital values into reflectance values for each of the pixels of the cropped image. Land cover spectral responses were determined by band math in the Red and Near-Infrared spectra, giving a normalized difference vegetation index (NDVI) raster image as a result. The NDVI image was subjected to an unsupervised classification by the Isodata method so as to obtain the different classes resulting from the spectral responses of the land cover present in the area of study [35,36]. The classification ended in 20 classes with 98% of convergence. By cluster analysis, pixels were grouped in 6 categories: Water, Uruguay River, Bare Soil, Urban Cover (includes non-paved streets), Low Density Vegetation, and Medium to High Density Vegetation. For each trap, a circular buffer area of 50 m was defined in order to avoid superposition, and the percentage of each class of land cover was calculated.

At each domestic unit, a set of 6 variables were recorded at the same time of the entomological sampling (*Trees*, *Fruit trees*, *Plant pots*, *Dogs*, *Hens* and *UnMat)* (Table 1). Variables as *Stream* and *Border*, were obtained from the satellite image and its posterior analysis by GIS. The ‘*Altitude*’ was recorded from the GPS at each trap position.

**Table 1:** Environmental variables used to explain the variation in *Lu*. *longipalpis* abundance at Santo Tomé, Corrientes.

Variable	Description	Habitat
*Dogs*	Number of dogs of the household	Micro
*Fruit trees*	Number of fruit trees of the household	Micro
*Hens*	Number of hens and chickens of the household	Micro
*Plant pots*	Number of plant pots of the household	Micro
*Trees*	Number of trees in a 10x10m quadrat around the trap	Micro
*UnMat*	Covered surface with unused materials (m^2^)	Micro
*Altitude*	Meters (extracted from GPS)	Macro
*Bare Soil cover*	Proportion of bare soil cover ^a^	Macro
*Border*	Distance from the trap to the nearest city border (km)	Macro
*Stream*	Distance from the trap to the nearest stream shore (km)	Macro
*HMDenVegC*	Proportion of high and medium density vegetation cover^a^	Macro
*LDenVegC*	Proportion of low density vegetation cover ^a^	Macro
*Urban cover*	Proportion of urban cover ^a^	Macro

^a^ measured in a 50 m buffer area around the trap.

### Statistical analysis

#### Sandfly presence and abundance

We calculated the accumulated abundance of *Lu*. *longipalpis* during the 3-night trapping period. Pearson correlation coefficients for the 13 variables were below 0.5, except for *Urban cover* with *LDenVegC* and *HMDenVegC* (-0.79 and -0.8 respectively). Also, variance inflation factors (VIF) were calculated with package *car* for *R* [37] showing very high values for *Urban cover*. When this variable was set aside, all VIF values were between 1 and 3 units. Therefore, *Urban cover* was not included in the models.

### Models

We constructed a set of 13 models to account for micro- (2), macro- (2) and mixed-habitats effects (9) (Table 2). Two models took into account all the measured variables after checking for collinearity (***NB full***, ***Hurdle full***). Ten models set aside the ‘animal’ variables (*Dogs*, *Hens*), because of its moving nature in contrast with the other ‘sessile’ things measured. As it was stated in the introduction, according to a conceptual framework that discriminates instrumentally spatial scales, conceptually the presence from the abundance phenomena, and allow to introduce the expert knowledge in the final models, 2 Hurdle models were constructed as an abundance part with 6–4 variables, and a presence part with 6 variables (***Hurdle micro/macro***, ***Hurdle micro sessile/macro***, respectively). Two models took into account a possible quadratic relationship of *Stream* with sandfly abundance, and the *number of trees at the trap* to represent shade and humidity at the microscale. Two other hurdle models were constructed only with biotic variables, excluding *Unused materials*, *Altitude*, *Border* and *Stream* (*Hurdle biotic 1*, both parts; *Hurdle biotic 2*, only count part). *Bare soil* was not considered in *Hurdle Biotic 2* since Bare Soil class had very low cover values in the entire city and could have a low influence in vector abundance/presence.

**Table 2:** Candidate models, variables included and habitat/s.

Type	Model	Variables	Habitat
**GLM Negative Binomial**	**Full**	Altitude + Bare Soil cover + Border + Dogs + Fruit trees + Hens + HMDenVegC + LDenVegC + Plant pots + Stream + Trees + UnMat	Mixed
	**Micro-sessile**	Fruit trees + Plant pots + Trees + UnMat	Micro
	**Macro**	Altitude + Bare Soil cover + Border + HMDenVegC + LDenVegC + Stream	Macro
	**Biotic**	Fruit trees + HMDenVegC + LDenVegC + Plant pots + Trees	Mixed
	**Shade/humidity**	Stream + Stream ^2^ + Trees	Mixed
**Hurdle model**	**Full**	Altitude^a^ ^,^ ^b^ + Bare Soil cover^a^ ^,^ ^b^ + Border^a^ ^,^ ^b^ + Dogs^a^ ^,^ ^b^ + Fruit trees^a^ ^,^ ^b^ + Hens^a^ ^,^ ^b^ + HMDenVegC^a^ ^,^ ^b^ + LDenVegC^a^ ^,^ ^b^ + Plant pots^a^ ^,^ ^b^ + Stream^a^ ^,^ ^b^ + Trees^a^ ^,^ ^b^ + UnMat^a^ ^,^ ^b^	Mixed
	**Micro-sessile**	Fruit trees^a^ ^,^ ^b^ + Plant pots^a^ ^,^ ^b^ + Trees^a^ ^,^ ^b^ + UnMat^a^ ^,^ ^b^	Micro
	**Macro**	Altitude^a^ ^,^ ^b^ + Bare Soil cover^a^ ^,^ ^b^ + Border^a^ ^,^ ^b^ + HMDenVegC^a^ ^,^ ^b^ + LDenVegC^a^ ^,^ ^b^ + Stream^a^ ^,^ ^b^	Macro
	**Micro/Macro**	Altitude^b^ + Bare Soil cover^b^ + Border^b^ + Dogs^a^ + Fruit trees^a^ + Hens^a^ + HMDenVegC^b^ + LDenVegC^b^ + Plant pots^a^ + Stream^b^ + Trees^a^ + UnMat^a^	Micro/Macro
	**Micro-sessile /Macro**	Altitude^b^ + Bare Soil cover^b^ + Border^b^ + Fruit trees^a^ + HMDenVegC^b^ + LDenVegC^b^ + Plant pots^a^ + Stream^b^ + Trees^a^ + UnMat^a^	Micro/Macro
	**Biotic 1**	Bare Soil cover^a^ ^,^ ^b^ + Fruit trees^a^ ^,^ ^b^ + HMDenVegC^a^ ^,^ ^b^ + LDenVegC^a^ ^,^ ^b^ + Plant pots^a^ ^,^ ^b^ + Trees^a^ ^,^ ^b^	Mixed
	**Biotic 2**	Altitude^b^ + Border^b^ + Fruit trees^a^ + HMDenVegC^a^ ^,^ ^b^ + LDenVegC^a^ ^,^ ^b^ + Plant pots^a^ + Stream^b^ + Trees^a^	Mixed/Macro
	**Shade/Macro**	Altitude^b^ + Border^b^ + HMDenVegC^b^ + LDenVegC^b^ + Stream^a^ ^,^ ^b^ + Stream^**2**^ ^a^ + Trees^a^	Mixed/Macro

For Hurdle models:

^a^Abundance part,

^b^Presence part.

#### NB models

The association between *Lu*. *longipalpis* accumulated abundance and the environmental characteristics related to each domestic unit was analyzed by a multiple regression procedure using GLM with a logarithm link function. The nature of the variable indicated a Poisson error structure, but to account also for high over dispersion we applied a Negative Binomial error structure [38]. Model parameter estimates where calculated by means of iteratively weighted least squares (IWLS), and maximum likelihood for *theta* using package *MASS* for *R* [39–41].

#### Hurdle models

We used a hurdle count regression model to predict the presence and the accumulated abundance of sandflies in a site as a function of explanatory variables measured at micro and macrohabitat [42,43]. The model has two components: a truncated count component for positive counts (with negative binomial distribution and *log* link), and a hurdle component for zero counts (with binomial distribution and *logit* link). With this approach, we can model simultaneously both the probability of occurrence and the abundance of sandflies, and search for environmental variables that may determine the presence and absence of the vector and/or the number of sandflies observed at each site.

### Model selection

The final set of candidate models was selected by means of the AICc criterion and taking into account the *Akaike weights* (*w*_*i*_, model probabilities) and ΔAICc [44–47]. Models with the lowest AICc and highest *w*_*i*_ were considered the best models in the set. Spatial autocorrelation in the raw variable and models residuals were checked by Moran’s I and semivariograms with *SAM software* [48]. Parameter estimates and BCa intervals (bias-corrected and accelerated bootstrap) of the final model(s) where calculated by bootstrap based on 1000 replications with package *boot* for *R* [49].

### Model diagnostics

To evaluate the predictive ability of the final model for the “presence part” we calculated: Kappa index, proportion of correct classifications (PCC), area under the curve (AUC), sensitivity and specificity with package *PresenceAbsence* for *R* [50]. As a threshold probability must be selected to distinguish positive from negative (sandfly presence and absence, respectively) all possible cut-off points from 0.01 to 0.99 were assessed to select an optimum cut-off point which maximized the Kappa index that assesses the improvement of classification of the model over chance.

### Canine leishmaniasis cases analysis

In first place, we analyzed the variable r*k39 positivity* (dichotomic, = 1 if dog had a positive rk39) by means of a generalized linear mixed model taking into account the clusters (random factor) made up of *Dogs house* plus *Dogs neighbors*. We constructed 5 models with *binomial family* and *logit link* using package *lme4* for R [51]. ***Model 1*** took into account individual dog characteristics such as: *breed*, *gender*, *age*, *size*, and *sterilization*; ***Model 2*** accounted for dogs habits: *night resting place*, *unleashed*, *moving history*, *repellent use*, *repellent periodicity*. ***Model 3*** included all the variables. ***Model 4*** was similar to model 1 but incorporating two interactions: gender*sterilization, and breed*sterilization. Models were compared by AICc.

In second place, we analyzed the association between the proportion of dog positivity in each trapped house and its neighbours *(Proportion of Positives*) and the centered environmental variables, including also the accumulated abundance of phlebotomines. Due to over dispersion, we constructed 5 GLM models with negative binomial family and log link (*variable*: number of positive dogs, *offset*: number of dogs analyzed) using the same variables as the ones listed as NB models in Table 2 and incorporating the accumulated abundance of *Lu*. *longipalpis* at each house, using package *MASS* for *R* [39].

## Results

### Entomological

**Fig 1:**
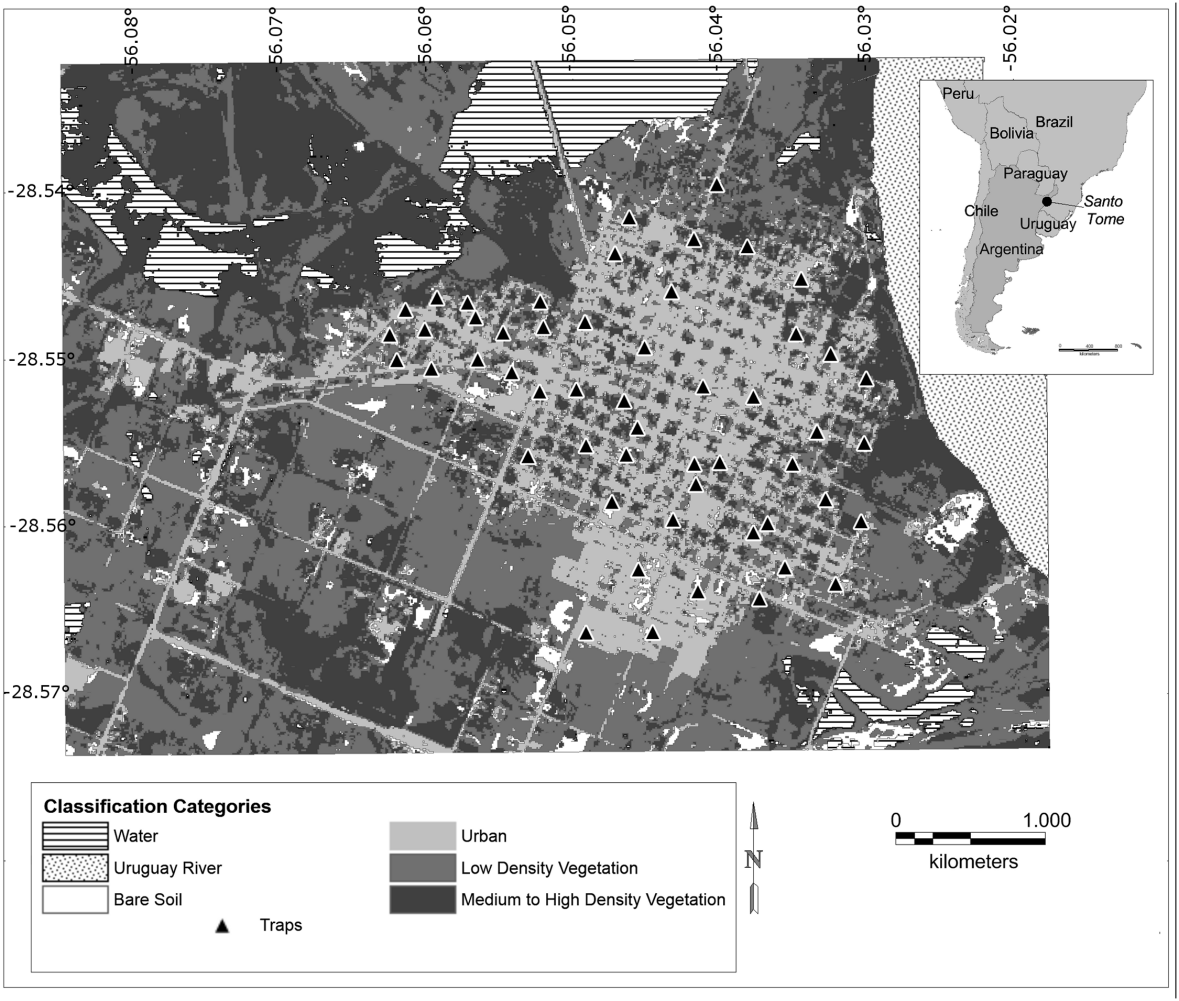
Spatial distribution of the proportion of dogs and sandfly. Spatial distribution of the proportion of rK39+ dogs (a) and sandfly accumulated abundance (b). Circle size represents the arbitrarily categorized values of the proportion of rK39+ dogs, and abundance of *Lu*. *longipalpis*, respectively. In parenthesis, number of sampling sites are indicated.

We captured a total of 853 sandflies belonging to six species: *Lu*. *longipalpis*, *Migonemyia migonei*, *Nyssomyia whitmani*, *Brumptomyia* sp., *Ny*. *neivai* and *Ev*. *cortelezzii*-*sallesi* (Table 3). The 98.35% of the sandflies captured were *Lu*. *longipalpis*. The capture effort was 53 traps/night (total: 159 traps), of which 51% were positive for *Lu*. *longipalpis* (Fig 1). Of this percentage, 85% were sites with abundances between 1 to 29 specimens, and 15% showed abundances higher than 30 individuals.

**Table 3:** Phlebotominae fauna by species and sex.

Species	Male	Female (gravid)	Total	%	Male/Female ratio
*Lu*. *longipalpis*	734	92 (13)	826	98.33	8/1
*Mg*. *migonei*	3	5 (1)	8	0.95	0.6/1
*Ny*. *whitmani*	0	2	2	0.24	-
*Ny*. *neivai*	1	0	1	0.12	-
*Ev*. *cortelezzii-sallesi*	0	1	1	0.12	-
*Brumptomyia sp*.	1	1	2	0.24	-
**Total**	739	101 (14)	840	100	7.3/1

### Canine

We sampled a total of 197 dogs, 177 of which were associated to households were insects were sampled (Fig 1). The rest of the dogs belonged to houses that could not be included in the insect sampling due to logistical issues. Positive rK39 dogs represented 16.75% of the total, of which 47% were asymptomatic. We did not find evidences of association between *rK39 Positivity* and the explanatory variables. The models showed no improvement compared to the null model.

As for the *Proportion of positives*, it seems to be associated with microscale variables such as *Trees near the trap* (p = 0.005) and *Stream* ^*2*^ (p = 0.008) but the effect could not be confirmed due to computational issues during bootstrapping.

### Environmental variables

#### Data extracted from satellite images

**Fig 2:**
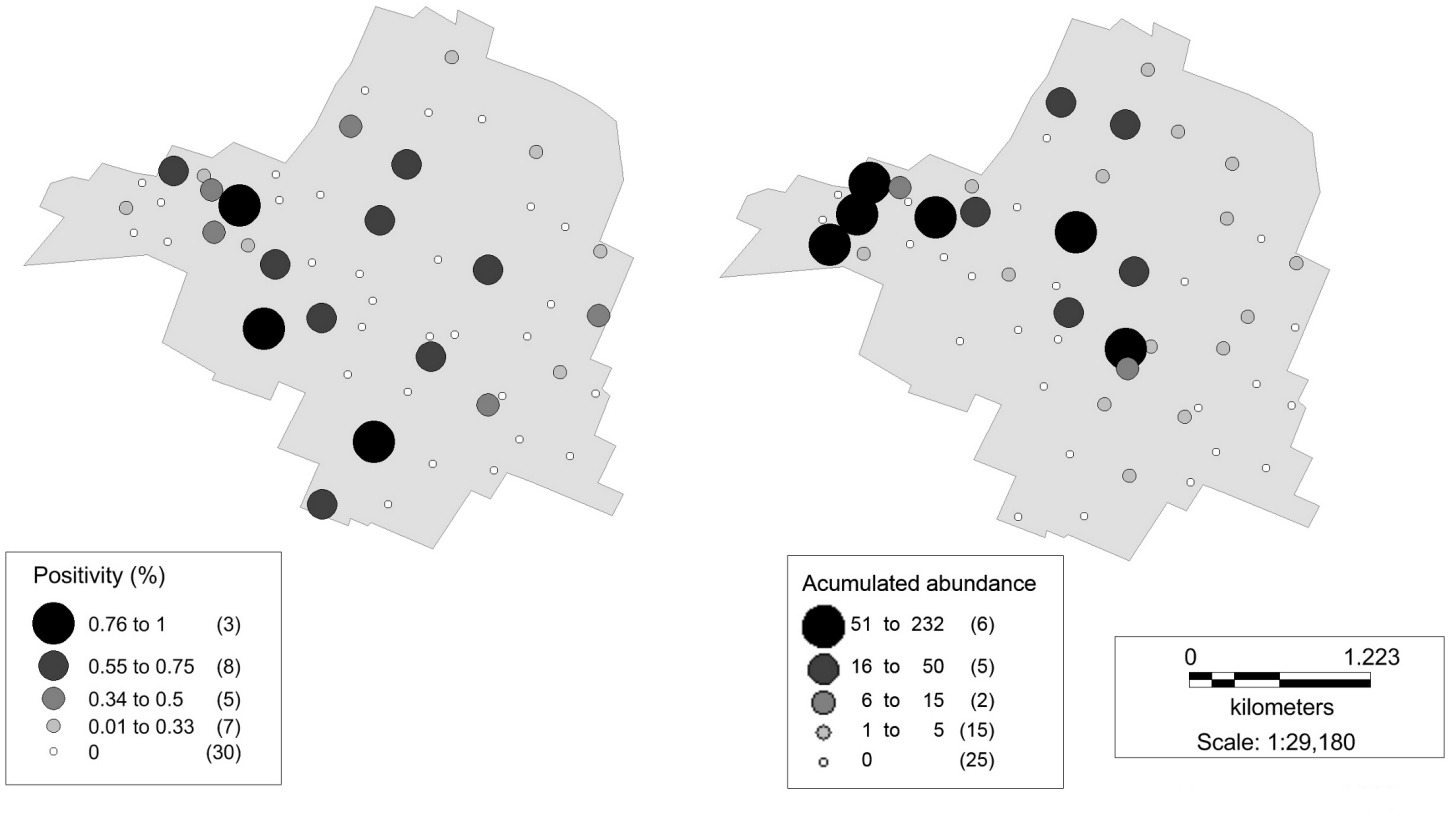
Land cover classification of the city. Land cover vectorial obtained after a non-supervised classification of the NDVI raster image. Sand-fly sampling sites are represented by the ▲ symbol. Areas of 50 m radius around the trap were used to calculate each type of cover proportion.

After the image classification of the land covering, the total area of the city was divided in 46.66% of *Urban cover*, 31.15% of *Low density vegetation cover*, 17.91% of *Medium to high density vegetation cover*, and 4.25% of *Bare soil cover* (Fig 2).

### Estimated models

After model selection, one NB model and three hurdle count regression models were responsible for 99% of the collective model weight (S1 Table). But the best model of the set was the *Hurdle shade/Macro* model that differed in almost 10 units (or more) of AICc from the others. After removing two non-significant terms from the presence part (BCa intervals contained the 0 value), *Altitude* and *Stream* ^*2*^, AICc diminished 4 units and this model was retained.

The Kappa index calculates the agreement between model predicted values and observed data, indicating how much better from a random classification the model is. The reduced final model had an intermediate Kappa value of 0.43 (SD = 0.12; optimum cutoff = 0.54). The reduced model improved the sensitivity from 0.55 (SD = 0.09) to 0.71 (SD = 0.08) but reduced the specificity from 0.91 (SD = 0.06) to 0.73 (SD = 0.1). The reduced final model correctly classified 72% (SD = 0.06) of the data (AUC = 0.75 (SD = 0.07)) (S1 Fig).

After calculating BCa confidence intervals for each estimate, only distance to the border of the city (*Border*) and high to medium density vegetation cover (*HMDenVegC*) ended to be explanatory, all positive, of the presence of sandflies in the city (Table 4). All variables in the abundance model ended to be explanatory, trees around the trap (*Trees*), distance to the stream and its quadratic (*Stream*, *Stream* ^*2*^), being the last one the only one with negative coefficient indicating that the maximum abundance was associated to medium values of distance to the stream.

**Table 4:** Parameters of the final model.

	Covariate	Parameter estimate	SE	Lower BCa CL	Upper BCa CL	P value
**Presence model**	Intercept	0.210	0.427	-0.637	0.991	0.562
	***Border***	**5.355**	**2.124**	**1.205**	**8.173**	**0.0036**
	***HMDenVegC***	**8.435**	**4.685**	**0.410**	**16.136**	**0.0194**
	*LDenVegC*	7.172	3.498	-0.187	12.992	0.0255
	*Stream*	3.282	2.062	-0.571	7.115	0.0531
**Abundance model**	Intercept	3.289	3.151	-8.495	4.302	<0.001
	***Stream***	**7.384**	**5.049**	**1.979**	**24.520**	**0.0016**
	***Stream*** ^***2***^	**-50.137**	**19.175**	**-80.91**	**-17.97**	**<0.001**
	***Trees***	**0.400**	**0.208**	**0.123**	**0.909**	**0.0014**

Bootstrapped parameter estimates of the reduced final model, *Hurdle Shade/Macro*, bootstrap SE values, and 95% BC a confidence limits (CLs) for covariates predicting the presence (binomial model) and abundance (count model) of sandflies in the city of Santo Tomé. Bold covariates do not include 0 in their CLs.

## Discussion

In Santo Tomé, the spatial distribution of dogs infected with *L*. *infantum* show a heterogeneous pattern throughout the city. We could not confirm an association of the distribution of infected dogs with the variables assessed. Although both dog’s positivity and vector abundance were found related to microhabitat variables we could not link them in this study. Besides environmental factors related to vector distribution, positive dog′s spatial pattern could be due to social factors, as networks of breeding or selling puppies (horizontal and vertical transmission), transit or traffic within the locality or with other endemic locations [13,52]. Indeed, similar results were reported in studies performed in different cities of Brazil, where higher concentrations of VL canine cases incidence were associated just with VL human cases or altitude [53–56]. However, a meta-analysis of the factors associated with canine VL in Brazil reported evidence of statistical association with one environmental variable (*presence of green areas adjacent to the house*), individual variables such as *short hair* and *pure breed*, and individual management variables (*peri-domestic/domestic restricted dogs*), but the authors also highlighted design and analysis limitations of the reviewed articles [57]. Also, besides the individual determinants and individual dog-management variables, other animal management variables related to attractiveness or dilution effect of blood sources for vectors were associated with dog seropositivity (*positive association with the number of cats in the households*, *protective presence of chickens and pigs*) [29].

This lack of strong or consistent associations in the literature could be related mainly to: a) design limitations due to work with: reported cases vs. actual incidence of infection, prevalence of past transmission vs. current environmental variables, individual factors of susceptibility-vulnerability-exposition mixed with environmental variables, dogs with different roaming area; b) inconsistencies between the spatial scales of dependent and explanatory variables; c) diagnosis limitations, in our study the majority of the rK39+ dogs were clinically asymptomatic, and it is known the relative low sensitivity of rK39 test in asymptomatic dogs [58,59]; and d) dog management practices, as the dog spatial distribution could be more associated with dog transit and puppies adopting (social/commercial networks of pets) than to the actual distribution of the probability of transmission [13,52]. The last point is even further important when at higher time-space scales the data from dogs in rural-periurban and urban landscapes are analyzed together.

We report *Ny*. *whitmani* for the first time in the study area. This species has been incriminated in the cutaneous leishmaniasis outbreaks due to *Leishmania braziliensis* of the Argentinean northeastern border both by natural infection and environment-time-space association with human cases, though observed abundances in the study area are still far from epidemic risk and this species has usually been associated to primary vegetation in Argentina [60,61]. However, it has been related to more urbanized environments in recent studies in the northeastern region [5].

In relation to *Lu*. *longipalpis* distribution, the strategy to discriminate the micro-spatial scales at which the environmental variables were recorded allowed us to associate presence with macrohabitat variables, and abundance with microhabitat and macrohabitat variables.

The presence of *Lu*. *longipalpis* was positively affected by the variables *Distance to the city border* and *High density vegetation cover*. As the distance to the city border increased, the probability of *Lu*. *longipalpis* presence tend to be higher. The variable *High density vegetation cover* showed also a positive relation with the vector presence. It can be explained by the generation of enabling environments for the presence of *Lu*. *longipalpis*. Though these variables seem to be contradictory, the city under study has a not uniform physiognomy presenting centric areas with high proportion of green surface, offering small breeding and resting conditions for the vector (Fig 2). The preference of *Lu*. *longipalpis* for complex urban environments [62] with green patches (between ruralized periurban and downtown) were reported in the literature [5,20,23,25,63,64]. Further, in cities as Rio de Janeiro, Brazil, *Lu*. *longipalpis* was found in Caju Cementery surrounded by highly urbanized blocks [65]. On higher spatial scales it was also observed the association of *Lu*. *longipalpis* and its sibling species *Lu*. *cruzi* with highly urbanized areas and low NDVI indexes, but with transitional and vegetation-patched landscapes [66–68].

The abundance of *Lu*. *longipalpis* showed association with variables at both types of scale. At the microhabitat level, the *number of trees around the trap* was positively related with the vector accumulated abundance. Trees offer a micro environment where *Lu*. *longipalpis* can find appropriate refuge; suitable breeding places [21] by means of physical properties (trunk structure, shadow size and quality); semiochemicals (the involved species could also be important) [23]; and tree coverage (100 m buffer) that showed an association with the abundance of this vector [25]. Other two variables that positively accounted for the differences in the vector’s abundance in the city were *distance to the water course* and its *quadratic*, both at the macrohabitat scale. Those areas placed at medium distances, between 470 and 710 m from the water course, showed an association with high abundances of *Lu*. *longipalpis*. On the other hand, houses outside this range had lower abundances. This result might indicate that water courses provide an optimum ‘window’ of humidity for the vector reproduction/survival, or for sandlfly predators (i.e. Scenopinidae larvae [69]), or might be also associated with the intermediate environmental heterogeneity between highly urbanized and rural landscapes. Santini *et al*. [23] found association of *Lu*. *longipalpis* abundance in urban scenarios with this variable also at microhabitat scale. On the other hand, in a study that used NDWI (Normalized Difference Water Index) and NDVI no correlation was observed with *Lu*. *longipalpis* abundance [22], showing again the importance about the consistency between the spatial scales of the hypothesis-sampling design and the conclusions.

Other variables once reported as associated with *Lu*. *longipalpis* presence or abundance did not show association in our study. The attractiveness of mammals and birds, mainly chickens, and its capacity to enhance breeding sites (moisture, manure, shadowed dwellings) was proposed [17,69], while the presence of chickens, but not its quantity, was associated with the abundance of *Lu*. *longipalpis* in urban settings [20]. The hen houses are usually a preferred site, selected by researchers and control programs to locate traps, as it is reported in the Materials and Methods section of many articles about *Lu*. *longipalpis* even this; therefore the homogeneity of this variable between trapping points at micro-scale could have masked the results, and the effect at the macrohabitat level was not measured. Low socio-economic level and poor sanitation (sewage system and rubbish collection) were associated with VL incidence and these associations were explained by vector suitability [18], although the facts beneath the increased vector exposition could be indicators of a more complex social determination of the disease distribution.

Considering the low temperatures registered during the sampling nights, sites with high trap positivity could reveal stable vector hot spots as the ones described at the city of Posadas in the 2007 and 2009 [20,23,25,64], while null sandfly traps could also be false negatives. The authors suggested that this stable sites with high abundance of *Lu*. *longipalpis* could act as source populations in a metapopulation structure within a ‘city network’ of connected patches. Therefore, to identify the sites in each scale and the variables associated with presence and abundance could contribute to assess the significance of particular habitat patches [70], with implications in vector control-surveillance integrated strategies [71,72]. At microscale, the operational questions to be answered will be for example, which households/areas within the city require specific interventions/recommendations at a given point of time? In this sense, to develop a model that explains more than 70% of the *Lu*. *longipalpis* distribution could contribute to propose environmental management control interventions. From individual practices to county planning (microhabitat to macrohabitat) the recommendations on density and species of trees, and potential breeding sites could be assessed experimentally. On the other hand, finding areas more suitable for *Lu*. *longipalpis* (hosting the populations with highest abundances in the village (distance to stream) by itself or as surrogate of socio-economic conditions or related practices (chicken breeding)), may be used to focus the allocation of resources, or to select the sites to evaluate the interventions.

In conclusion, discriminating environmental spatial based variables recorded at mesohabitat and microhabitat buffers and modeling *Lu*. *longipalpis* presence and abundance as different components, allowed to explain 70% of the vector presence. Based on the variables associated with *Lu*. *longipalpis*, the model will be validated in other cities and environmental surveillance and control interventions will be proposed and evaluated in the microscale level. In this sense, programmatic and village strategies integrated with socio-cultural approaches could be incorporated in city, neighborhood and individual environmental management, according to each mesoscale and microscale scenarios, based on participatory action methodologies, so the actual intervention will be defined together with community [73].

## Supporting Information

S1 Table Model selection metrics of models. Model selection metrics for NB and hurdle count regression models fit to presence and/or abundance data for Phlebotominae sandflies at 53 sites. Model results are ranked by AICc from best to worst.
(DOC)

S1 Fig Probability of sandfly presence. Estimated probability of sandfly presence (dots and smoothed line) in terms of increasing proportion of vegetation cover (Low + Medium/High density vegetation cover proportion). ID: trap/site.
(TIF)
