# Correction: *Lutzomyia longipalpis* Presence and Abundance Distribution at Different Micro-spatial Scales in an Urban Scenario

**DOI:** 10.1371/journal.pntd.0004091

**Published:** 2015-09-25

**Authors:** The PLOS Neglected Tropical Diseases Staff

The images for Figs 1 and 2 are incorrectly switched. The image that appears as Fig 1 should be Fig 2, and the image that appears as Fig 2 should be Fig 1. The figure captions appear in the correct order.
