# Supplementary material for: Lutzomyia longipalpis Presence and Abundance Distribution at Different Micro-spatial Scales in an Urban Scenario
Source: PLoS Negl Trop Dis. 2015 Aug 14;9(8):e0003951. doi: 10.1371/journal.pntd.0003951 (PMC4537120; doi:10.1371/journal.pntd.0003951)
Supplement: S1 Table — Model selection metrics for NB and hurdle count regression models fit to presence and/or abundance data for Phlebotominae sandflies at 53 sites. Model results are ranked by AICc from best to worst. (DOC) [file pntd.0003951.s001.doc]

**Supporting Information**

**S1. Model selection metrics of models: Model selection metrics for NB and hurdle count regression models fit to presence and/or abundance data for Phlebotominae sandflies at 53 sites. Model results are ranked by AICc from best to worst.**

| **Model** | **k** | **L-L** | **AICc** | **ΔAICc** | **w_i_** |
| --- | --- | --- | --- | --- | --- |
| Hurdle Shade/Macro | 12 | -116 | 263 | 0,0 | 0,99 |
| Nb Shade/humidity | 5 | -131 | 273 | 9,8 | 0,01 |
| Hurdle Biotic 2 | 13 | -122 | 279 | 16,3 | 0,00 |
| Hurdle Macro | 15 | -119 | 280 | 17,6 | 0,00 |
| Nb Biotic 2 | 7 | -133 | 283 | 20,3 | 0,00 |
| NB Micro sessile | 6 | -135 | 284 | 20,8 | 0,00 |
| Hurdle Micro sessile/Macro | 13 | -125 | 285 | 21,9 | 0,00 |
| NB Macro | 8 | -134 | 287 | 23,8 | 0,00 |
| Hurdle Micro/Macro | 15 | -123 | 290 | 26,8 | 0,00 |
| Hurdle Micro sessile | 11 | -131 | 290 | 27,2 | 0,00 |
| NB full | 14 | -127 | 293 | 30,6 | 0,00 |
| Hurdle Biotic 1 | 15 | -125 | 294 | 30,8 | 0,00 |
| Hurdle full | 27 | -113 | 340 | 77,5 | 0,00 |
